# Modified abdominal advancement flap with or without the Ochikomanai method: Oncoplastic surgery using a simple volume replacement technique

**DOI:** 10.1007/s00595-026-03447-1

**Published:** 2026-07-31

**Authors:** Erina Hatakawa, Kaho Nakamura, Rena Yamakado, Miyuko Yoshikawa, Mai Shibusawa, Mao Kimoto, Nao Imai, Makoto Ishitobi, Kousuke Kawaguchi, Tomoko Ogawa

**Affiliations:** 1 Department of Breast Surgery, Mie University Graduate School of Medicine, Mie, 2-174 Edobashi, Tsu, 514-8507 Japan; 2 Department of Breast Surgery, Ise Red Cross Hospital, Mie, Japan

**Keywords:** Breast cancer, Breast-conserving surgery, Oncoplastic surgery, Reconstructive surgical procedure, Volume replacement technique

## Abstract

**Purpose:**

To evaluate the usefulness of modified abdominal advancement flap (modified AAF) with and without the Ochikomanai method for breast-conserving surgery (BCS) based on cosmetic results and patient satisfaction.

**Methods:**

We conducted a retrospective cohort study focusing on the usefulness of the modified AAF, based on cosmetic results and patient satisfaction, in 88 patients (89 breasts) who underwent BCS using the modified AAF with or without the Ochikomanai method. A historical cohort of patients who underwent standard AAF was used for comparison.

**Results:**

Ten patients (11%) had complications; however, no fat necrosis was observed, and all complications improved with conservative treatment. With regard to cosmetic results, 41 breasts (58%) were rated excellent, 26 (37%) were rated good, and only 4 ([6%]) were unacceptable, all of which were rated fair. Patients with medium- or larger-sized breasts and older patients who underwent BCS using the modified AAF had acceptable cosmetic results. More than 89% of the patients indicated that they were somewhat or very satisfied in all 8 categories, except for physical limitations in the immediate postoperative period.

**Conclusion:**

BCS using a modified AAF, with or without the Ochikomanai method, is a useful technique for many patients, including those who are older and those with relatively large breasts.

## Introduction

Breast-conserving surgery (BCS) remains the standard approach for treating early-stage breast cancer, balancing oncological safety with aesthetic preservation [1]. However, performing BCS in the lower quadrant of the breast frequently leads to suboptimal cosmetic outcomes [2]. To address this challenge, oncoplastic surgery techniques incorporating reduction methods originally developed for aesthetic plastic surgery began gaining popularity in Europe and the United States in the 1990s [3, 4]. In Japan, various methods, such as the downward moving procedure, have been considered to address this challenge [5].

Oncoplastic surgery encompasses two primary techniques: volume displacement and volume replacement. Volume displacement involves reshaping the breast using the remaining mammary tissue to fill resection cavities, whereas volume replacement utilizes extramammary tissue to reconstruct the defect following BCS [6]. Since most Japanese women’s breasts are too small to remodel with only a volume displacement technique, and because Japanese patients prefer not to undergo operation on the unaffected side, volume replacement techniques are preferred. Some volume replacement methods, such as the use of perforator flaps and latissimus dorsi myocutaneous flaps, have been reported [7, 8]. These flaps can be used to fill large defects. However, these techniques are complex and result in the formation of additional donor-site scars, which often preclude their use.

In response to the need for improved aesthetic outcomes in the lower breast region, particularly among Japanese patients, we implemented the abdominal advancement flap (AAF) approach in 2010. AAF provides a straightforward volume replacement option that can be effectively used by breast surgeons without requiring additional expertise in plastic surgery.

Initially, we expected that BCS with AAF would have better cosmetic results when used in younger patients, those with a lower body mass index (BMI), and those with smaller and non-ptotic breasts. However, in our previous study, we found that the percentage of unacceptable cases increased when AAFs were used in patients with a high BMI, large breasts, tumors located in the inner portion, and an older age [9]. In addition, AAFs are difficult to perform in patients at a high risk of impaired blood flow, such as older patients and those with diabetes, because the technique requires extensive skin flap creation. Therefore, there is a need to develop techniques that can be used in patients without AAF indications.

To fill this need, we developed an even simpler volume replacement technique based on the AAF in 2017 and named the technique “modified AAF.” Modified AAF is a technique for filling a defect using the skin and subcutaneous tissue of the upper abdomen and lateral chest and reconstructing the inframammary fold (IMF) simultaneously. To advance the filling tissue from the lateral and caudal sides, this technique was performed mainly in patients with tumors on the lateral and caudal sides. Because of the simplicity of the procedure, we expanded the indications for modified AAF. However, if the skin on the tumor is not removed during the filling process, the excess skin over the defect sinks in and wrinkles. Therefore, even if the breast shape is good, should the excess skin become sunken, the evaluation of cosmetic results is likely to decrease, and if the cosmetic results are poor, both the patient and the breast surgeon tend to feel disappointed in the outcome.

We therefore developed a simple solution to solve the problem of excess skin depression, named the “Ochikomanai method,” a play on the Japanese phrase “Ochikomanai,” which means both “does not sink” and “does not feel disappointed”. We applied the modified AAF with the Ochikomanai method in patients with extra skin over the defect. In the standard AAF procedure, the neo-IMF is anchored to the chest wall using absorbable sutures, and the defect left by the partial mastectomy is filled by mobilizing the surrounding mammary gland tissue. This typically requires a wide subcutaneous undermining from the caudal breast area to the original IMF. In contrast, the modified AAF technique anchors the neo-IMF to the cranial edge of the mammary gland, allowing the flap to simultaneously fill the defect without extensive subcutaneous dissection.

We herein report the use of the modified AAF with and without the Ochikomanai method for BCS and assess its usefulness based on cosmetic results and patient satisfaction.

## Methods

A retrospective cohort study was conducted to assess the effectiveness of BCS using a modified AAF, with or without the Ochikomanai technique, with a focus on cosmetic outcomes and patient satisfaction. In standard AAF, the skin flap is mobilized extensively and advanced from the caudal abdominal area, often requiring wide undermining of subcutaneous tissues. In contrast, the modified AAF requires a smaller dissection area and enables volume replacement with a more localized flap.

The study population comprised 88 patients (89 breasts) diagnosed with primary breast cancer or benign breast tumors who underwent BCS or lumpectomy using the modified AAF technique at Mie University Hospital between June 2017 and April 2022. All procedures were performed solely by breast surgeons, without the assistance of plastic surgeons. To evaluate the efficacy of the modified AAF technique, we compared our findings with previously published data on standard AAF performed at our institution. We previously reported the outcomes of 41 patients who underwent standard AAF, which served as the historical control group for this analysis [9].

Indications for BCS or tumorectomy with the modified AAF included lesions outside the upper inner quadrant. The modified AAF was used when the available tissue between the IMF and neo-IMF covered at least 80% of the resected volume. When excess skin remained after the procedure, the Ochikomanai method was applied to improve the contour. At our institution, skin excision directly over the tumor is routinely performed in many cases to ensure a good surgical field. However, this has not been uniformly applied. In cases where the tumor was distant from the skin, minimal skin excision was performed, or preserved skin was used as a landmark. Since the introduction of the Ochikomanai method, we aimed to preserve the skin and utilize de-epithelialization to fill the defect, thereby avoiding unnecessary excision.

The modified AAF technique was primarily applied to tumors located in the lateral and caudal portions of the breast, including advancement of tissue from the lateral chest wall. Therefore, tumors in the upper outer quadrant are generally considered good candidates. For tumors in the upper inner quadrant, the indication was limited; however, in select cases close to the nipple-areola complex (NAC), where the incision could be placed in an arc parallel to the IMF, this technique was successfully applied.

The estimated excised volume suitable for this procedure is approximately 25% of the total breast volume. Primary indications include tumors located in the lateral and caudal breast regions.

### Surgical techniques

**Fig. 1:**
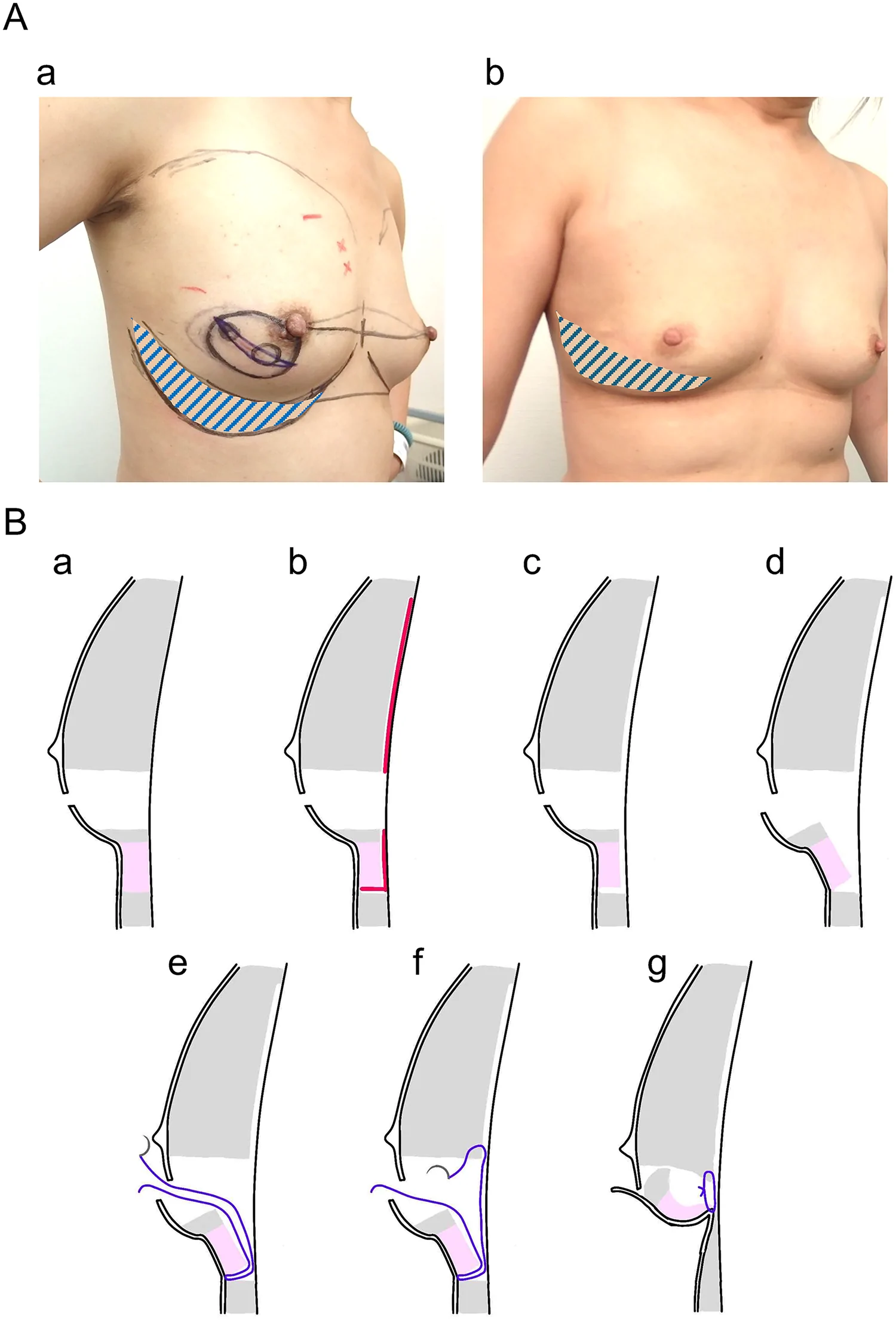
Schematic illustrations of the surgical procedure of the modified AAF. [**1-a**] Preoperative markings and postoperative condition. (**a**) Preoperative markings. The neo-IMF was marked such that it could be easily lifted to the height of the original IMF. The skin incision line was set parallel to the neo-IMF. The area between the IMF and neo-IMF was the upper abdominal and lateral thoracic tissue used for defect filling, as shown in pink. (**b**) Postoperative conditions. The range from the IMF to the neo-IMF was included in the breast and is shown in pink. [**1-b**] Vertical views of the procedures. (**a**) BCS was performed. (**b**) Extensive undermining of the mammary gland on the chest wall on the caudal side to the neo-IMF and on the cranial side. (**c**) By cutting the subcutaneous tissue of the neo-IMF, it became a sharp angle. (**d**) Absorbable interrupted sutures were inserted into the subcutaneous tissue of the neo-IMF in 1-cm width. (**e**) The sutures were threaded through the cranial mammary stamp. (**f**) The sutures were subsequently tied and fixed to the cranial mammary stamp. The neo-IMF was created by filling a defect in the partial resection and pulling the NAC down to the same height as the unaffected side. AAF: abdominal advancement flap; neo-IMF: new inframammary fold; IMF: inframammary fold; NAC: nipple-areola complex

The procedure for creating the modified AAF is illustrated (Fig. 1). Before the operation, the original IMF, neo-IMF, height of the nipple on the unaffected side, and skin incision line were marked, with the patient in the standing position. The neo-IMF was marked where the skin could easily be lifted to the height of the original IMF, and the position of the neo-IMF was approximately 3–5 cm below the IMF. The skin incision line was parallel to the neo-IMF incision line.

The incision line in the modified AAF procedure followed the same principles as in the standard AAF. The incision was placed along the tumor site and often parallel to the neo-IMF because of the tumor location. It was not located between the original IMF and neo-IMF. The neo-IMF was reconstructed approximately 3–5 cm below the original IMF, and this lower positioning allowed for improved breast contours and symmetry. When the tumor was close to the skin, the skin in the area close to the tumor was excised.

Subsequently, we performed BCS. We did not change the BCS procedure regardless of incorporation of the modified AAF, except for the skin incision. After BCS, the mammary gland on the chest wall on the caudal side was undermined until neo-IMF was reached. In standard AAF, a skin flap is created up to the original IMF, and the mammary gland on the chest wall side is undermined from the original IMF until the neo-IMF. Similarly, the mammary glands in the chest wall on the cranial side were extensively undermined. A slit was made close to the skin in the subcutaneous tissue of the neo-IMFs. Some absorbable sutures were inserted into the subcutaneous tissue of the neo-IMF at a 1-cm width. We also verified that the neo-IMF was smooth when traction was applied to the sutures. A sharp angle was formed by the slit of the neo-IMF. The sutures were then tied and fixed to cranial mammary tissue. Creating the neo-IMF using this procedure allows filling of a defect from partial resection and pulls the NAC down to the same height as that on the unaffected side.

The position of the advanced tissue was adjusted depending on the tumor location. For cranially extended defects, the tissue was advanced superiorly, while for medial defects, the lateral breast tissue was mobilized toward the center. This flexibility allows for optimal defect coverage while maintaining breast symmetry.

**Fig. 2:**
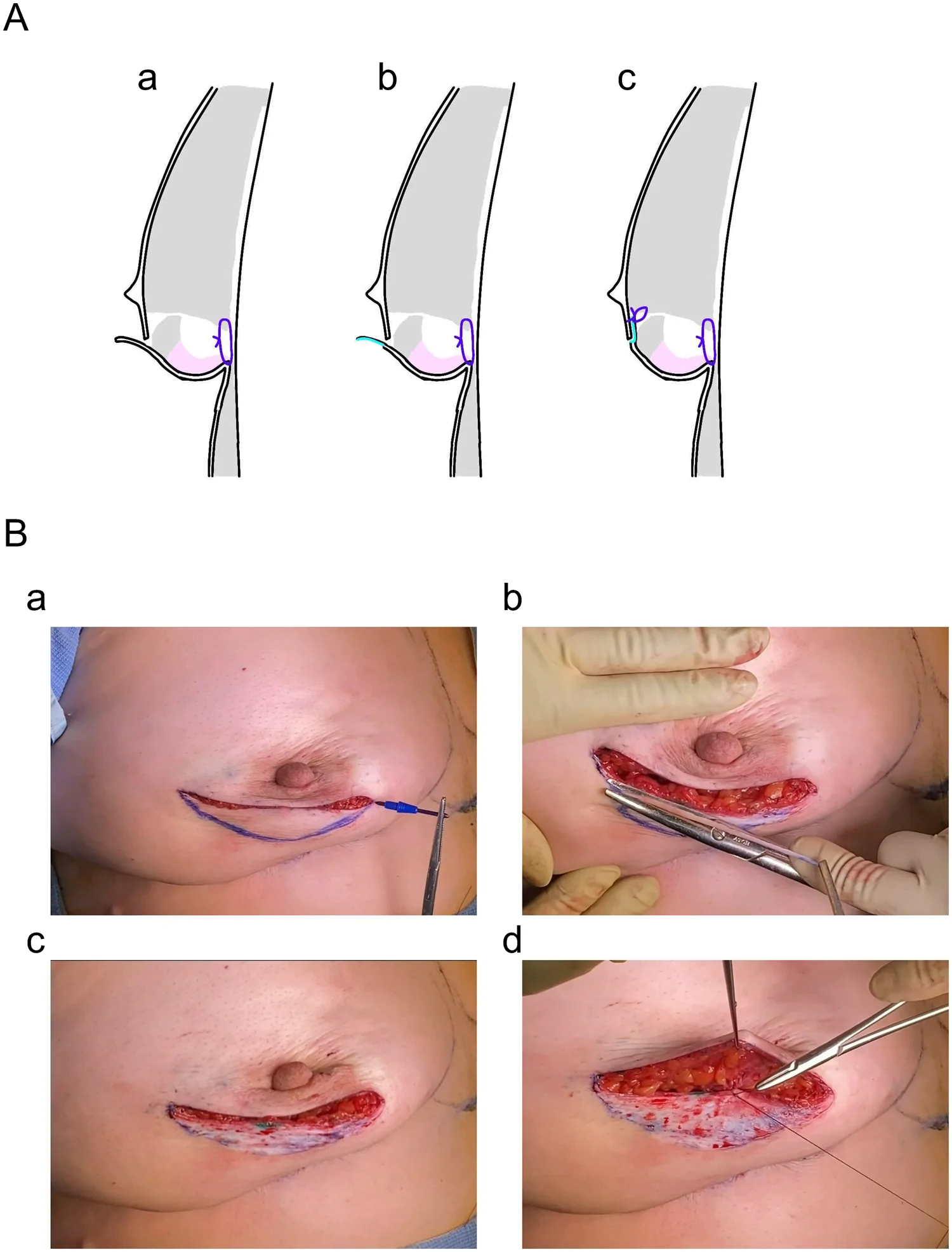
Schematic illustrations of the surgical procedure and operation photos of the Ochikomanai method. [**2-**a] Schematic illustrations of the Ochikomanai method surgical procedure. (**a**) The skin on the cranial side of the wound covered the skin on the caudal side of the wound; the skin on the caudal side of the overlapping area was marked. (**b**) The skin on the caudal side of the overlapping area was de-epithelized. (**c**) De-epithelized skin and upper mammary glands were sutured and fixed. [2-**b**] Operation photos of the Ochikomanai method. (**a**) The skin on the caudal side of overlapping area was marked. (**b**,** c**) The skin on the caudal side of the overlapping area was de-epithelized. (**d**) De-epithelized skin and upper mammary glands were sutured and fixed

If there was excess skin due to a small amount of skin resection on the tumor, we additionally performed the “Ochikomanai method” (Fig. 2). In cases in which skin excision resulted in excess skin relative to the volume of breast tissue resected, the Ochikomanai method was employed to prevent skin depression and ensure a smooth contour. However, the modified AAF technique itself remained unchanged, regardless of whether or not the Ochikomanai method was used. In the Ochikomanai method, de-epithelized excess skin props the skin over the defect and fills it at the time of skin closure. The skin on the cranial side of the wound covered that on the caudal side of the wound, and the skin on the caudal side of the overlapping area was de-epithelized. The de-epithelized skin and upper mammary glands were sutured and fixed. We decided to use the Ochikomanai method after BCS and the modified AAF if there was extra skin. Therefore, the BCS and modified AAF procedures do not depend on whether or not the Ochikomanai method is used.

The area to be de-epithelialized was a crescent-shaped area with the lower edge of the wound margin as the upper edge. In most cases, the width of the de-epithelialized area was 1–2 cm, which is the width of the excess skin after a modified AAF and varies from case to case.

In the modified AAF, the skin and subcutaneous tissue are advanced from the lateral and caudal sides of the resection to fill the defect; therefore, NAC is less likely to deviate in the direction of resection. As the nipple is less likely to deviate, there is no need to perform NAC re-centralization.

## Assessment of reconstruction

To assess the outcomes of reconstruction, a cosmetic evaluation was performed by seven breast surgeons, along with an evaluation of patient satisfaction.

The cosmetic evaluation was performed using photographs taken more than six months after the operation. We evaluated the cosmetic results of 71 patients who did not require reoperation and were photographed more than six months after the operation. Photographs of the patients’ breasts were taken in frontal, left oblique, and right oblique views without any identifying features. The cosmetic results were then evaluated by seven independent observers (breast surgeons) as “excellent,” “good,” “fair,” or “poor” using the Harvard Scale established by Harris et al. [10]. An excellent result indicated that the treated breast was nearly identical to the untreated breast; a good result indicated that the treated breast was slightly different from the untreated breast; a fair result indicated that there was an obvious difference between the two sides without major distortion; and a poor result indicated that the treated breast was seriously distorted. We classified excellent and good results as acceptable and fair and poor results as unacceptable. The observers were blinded to the identities of all the patients.

The patient evaluations were performed using a questionnaire. We administered questionnaires to 68 patients who were undergoing postoperative follow-up at Mie University Hospital, had not undergone residual mastectomy, and were able to complete the questionnaire in Japanese. The questionnaire was sent to target patients after confirming that it was acceptable to send the questionnaire at the outpatient clinic. The questionnaire included nine items: early postoperative physical limitations, current physical limitations, breast shape when dressing, breast shape when undressing, breast size, surgical scarring, whether they would recommend this surgery to others in the same situation, psychological resistance to public bathing, and comparison with preoperative expectations. For each item, patients rated their satisfaction on a 4-point scale.

## Results

The baseline characteristics of the study population are summarized in Table 1. Among the 88 patients, 18 (20%) were aged > 65 years and 22 (25%) had a BMI greater than 25 kg/m^2^. Breast sizes categorized as extra-small, small, or medium constituted 89% of the cases. Breast ptosis was assessed based on the Regnault classification, with 20% (17 patients) exhibiting some degree of ptosis. In addition, mammary gland density analysis revealed that 75% (67 breasts) were dense. Six patients had diabetes, and nine reported being smokers.

**Table 1:** Clinical characteristics of patients

Age (years)	*n* = 88
Average (range)	54.3 (28-81)
≥65 years	18
Body mass index, kg/m^2^	*n* = 88
Average (range)	22.9 (15.8-31.5)
≥25 kg/m^2^	22
Breast size	*n* = 85*
Extra small	9
Small	32
Medium	35
Large	5
Extra large	3
Breast ptosis	*n* = 85*
None	68
Grade Ⅰ	6
Grade Ⅱ	9
Grade Ⅲ	2
Breast density	*n* = 89
Dense breast	67
Scattered breast	22
Patients with diabetes mellitus	6
Smokers	9

* Breast size and ptosis were classified based on perioperative breast photographs. Therefore, only 85 breasts photographed perioperatively were included in this classification

Surgical data are presented in Table 2. The excision volume, compared with the total breast volume, was estimated using a preoperative photograph of the markings made for the partial resection area by seven independent observers (breast surgeons). The average excision volume relative to the breast volume was 16.6%. The excision volume percentage of the total breast volume was > 25% in 9 breasts (11%). The tumor was located in the outer upper or lower quadrant in 72 (81%) patients. Operative time and blood loss were examined in 68 patients, excluding those in whom surgery was performed on both sides of the breast, axillary dissection was performed, SNB was not performed, or intraoperative complications (hook wire straying) occurred. The mean operative time was 1 h and 45 min, and the mean blood loss was 14.7 g.

**Table 2:** Surgical data

Proportion of excision volume (%) *		*n* = 83
	5%	2
	10%	17
	15%	29
	20%	26
	25%	6
	30%	3
Location of the tumor		*n* = 89
	Central	7
	Inner upper	5
	Inner lower	5
	Outer upper	42
	Outer lower	30
Axillary treatment		*n* = 89
	None (breast surgery only)	11
	Sentinel lymph node biopsy only	69
	Blood loss**	*n* = 68
	Range (g)	0-46
	Average (g)	14.7
	Operation time**	*n* = 68
	Range (minutes)	61-164
	Average (minutes)	103
	Axillary dissection (including sampling)	9
Combining technique		*n* = 89
	Only mobilization (including the Ochikomanai method)	86
	Round block technique	1
	Modified round block technique	2
Nipple-areola complex excision		2
Additional surgery		*n* = 89
	No	77
	Yes (mastectomy)	8
	Yes (re-excision)	4
Postoperative complication ***		*n* = 81
	Total	10
	Treatment for complications	
	Reoperation due to complications	0
	Only conservative treatment	10
	Complication details	
	Wound infection	4
	Postoperative bleeding	3
	Delayed wound healing	6
	Wound dehiscence	3
	Nipple-areola complex/skin necrosis	3
	Intertrigo around the neo-IMF	1
	Fat necrosis	0

* Six breasts without preoperative photographs were excluded

** One patient with intraoperative trouble (hook-wire straying) was excluded

*** Eight patients who underwent mastectomy after BCS were excluded

Neo-IMF: new inframammary fold; BCS: breast-conserving surgery

Breast reshaping was achieved primarily through the modified AAF technique, with glandular flap mobilization applied to 86 breasts (97%), supplemented by the Ochikomanai method as needed. Concurrent round-block techniques were used in three cases (two patients), and NAC resection was necessary in two cases. In eight breasts, mastectomy was performed because of positive or close surgical margins, while re-excision was required in four breasts. Complications associated with the modified AAF occurred in 11% (10 patients) of patients; however, no cases of fat necrosis were reported, and all complications resolved with conservative management.

A comparative analysis between the standard AAF (historical control) and modified AAF is presented in Table 3. In our previous study, we reported that among 41 patients who underwent standard AAF, 26.8% had unacceptable cosmetic outcomes, with poorer results particularly observed in older patients and those with larger breasts [9]. In contrast, among the patients in the present study who underwent modified AAF, only 5% showed unacceptable cosmetic results. This suggests that modified AAF improves aesthetic outcomes, particularly in older patients and those with medium-to-large breasts, by reducing the risk of contour deformities and enhancing breast symmetry.

**Table 3:** A comparison with AAF

	AAF[8] (*n* = 41)	modified AAF (*n* = 71*)
Age (average, years)	53.3	54.3
BMI (average, kg/m^2^)	22.2	22.9
Breast size		
Extra-small	10 (24%)	7 (10%)
Small	22 (54%)	27 (38%)
Medium	9 (22%)	31 (44%)
Large	0	5 (7%)
Extra-large	0	2 (3%)
Excision volume (average)	21%	16.60%
Cosmetic result		
Acceptable	30 (73%)	67 (94%)
Age (average, years)	51.8	53.7
Unacceptable	11 (27%)	4 (6%)
Age (average, years)	57.2	54.2
Medium/Large/Extra-large breast	4 (44%)	2 (5%)

*Only patients for whom a postoperative cosmetic assessment was possible were considered for the comparison

AAF: abdominal advancement flap; BMI: body mass index

**Fig. 3:**
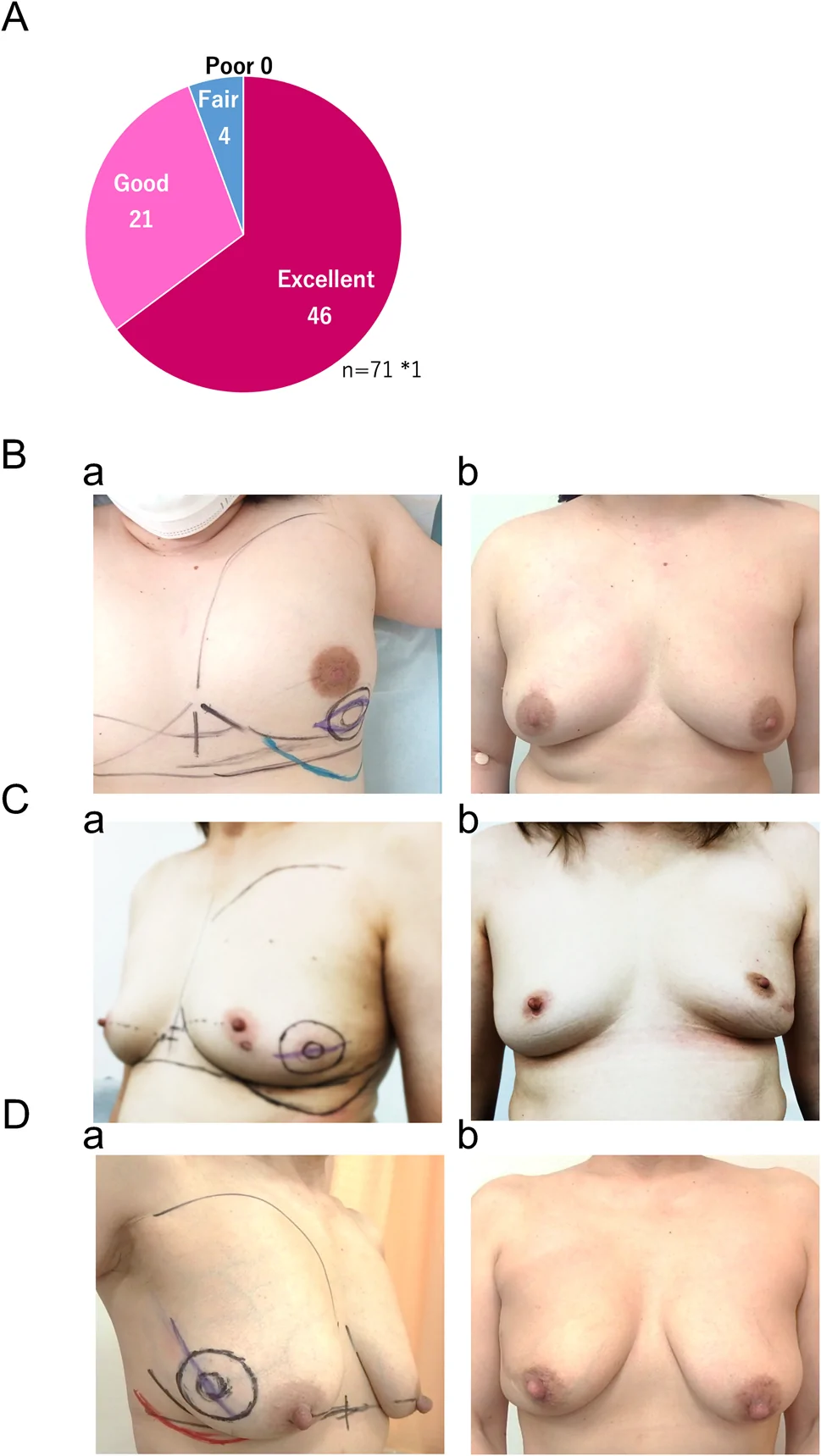
Cosmetic results. [**3-a**] Overall cosmetic results. We included only patients who did not require reoperation and were photographed more than six months after the operation. [**3-b**] A representative case of excellent results. The patient was 33 years old with a BMI of 27.3 kg/m^2^. Her breast was medium-sized. The excisional volume was 15%. (**a**) Preoperative markings. (**b**) Photographs taken one year and four months postoperatively. [3-c] A representative case of good results. The patient was 61 years old with a BMI of 24.7 kg/m^2^. Her breast was small-sized. The excisional volume was 15%. (**a**) Preoperative markings. (**b**) Photographs taken 1 year after the operation. [**3-d**] A representative case of good rating at cosmetic result. The patient was 53 years old with a BMI of 21.1 kg/m^2^. Her breast was medium-sized. The excisional volume was 15%. (**a**) Preoperative markings. (**b**) Photographs taken one year and two months postoperatively. The reason for the fair evaluation was the mild deviation of the nipple-areola complex. BMI: body mass index

The cosmetic evaluation indicated that 94% of the patients achieved satisfactory outcomes, with no cases receiving poor ratings. Specifically, 65% of the breasts (46 cases) were rated as excellent, 30% (21 cases) as good, and 6% (4 cases) as fair, with no instances of severe cosmetic deficits (Fig. 3).

**Fig. 4:**
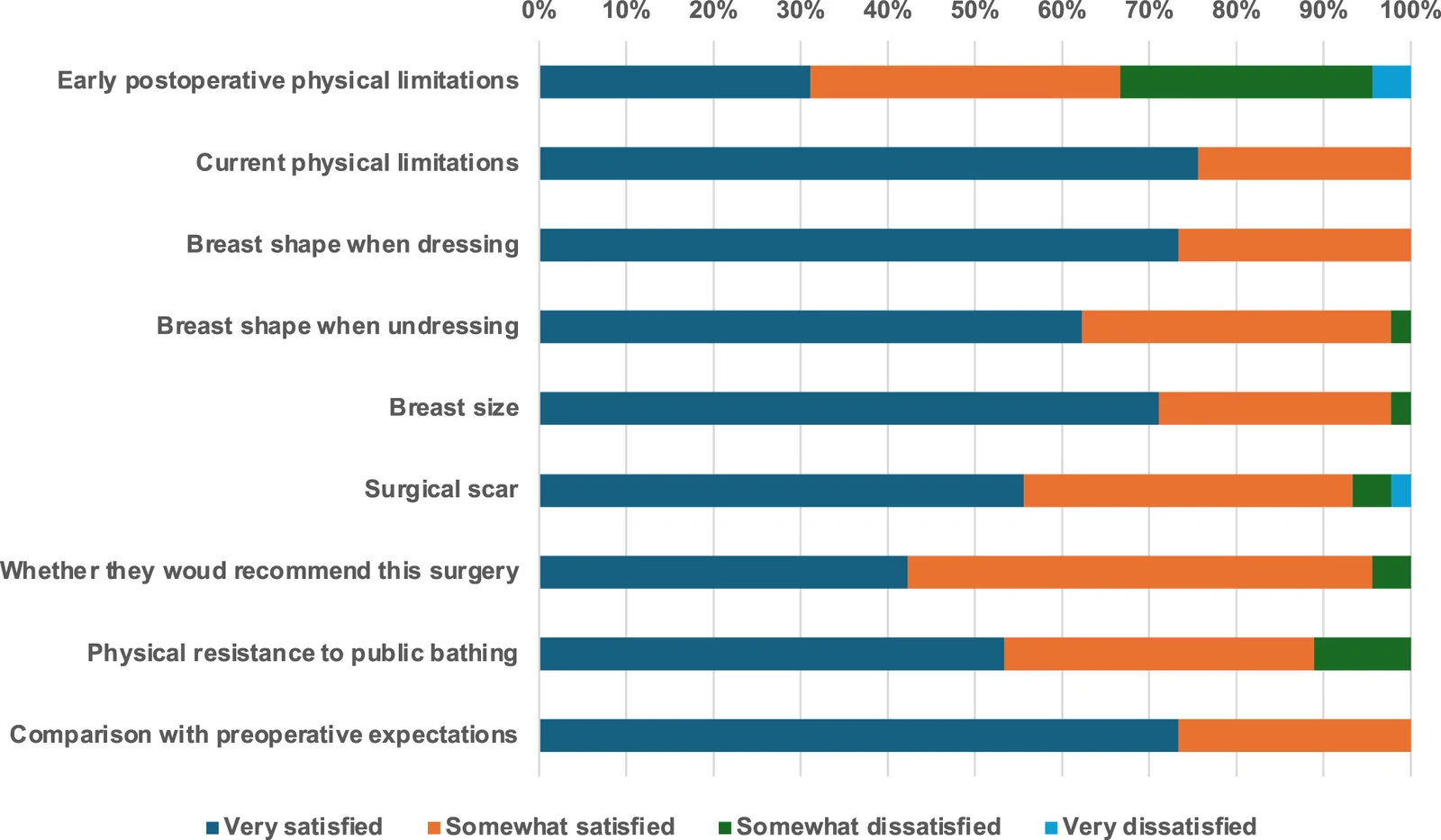
Patient evaluation results

Patient satisfaction data, as illustrated in Fig. 4, revealed that questionnaires were distributed to 68 patients, and 53 responses were received. Of these, 45 consented to using their responses for study purposes. Notably, 89% of the respondents expressed being somewhat or very satisfied across all 8 categories, excluding immediate postoperative physical limitations. In particular, all patients indicated that they were somewhat or very satisfied with their current physical limitations, breast shape when dressing, and comparison with preoperative expectations. 42 patients (93%) rated their satisfaction as either satisfied or generally satisfied with the 5 items that directly assessed cosmetic results.

Among the 89 breasts treated, the Ochikomanai method was used in 36% of cases (32 of 89 breasts), particularly in patients with excess skin after skin excision. This technique effectively prevented skin depression and improved aesthetic outcomes.

## Discussion

BCS targeting tumors in the lower breast often yield suboptimal cosmetic outcomes. Existing oncoplastic techniques for lower-breast tumors are typically designed for larger breasts and involve intricate procedures, which may not be feasible or appropriate for patients with smaller breasts, such as those commonly used in the Japanese population. Consequently, there is a pressing need for a streamlined, accessible technique tailored to lower-portion breast cancers in patients with small to medium-sized breasts. In 2010, we initiated BCS using AAF for breast cancer of the lower portion of the breast. However, in our previous study [8], 26.8% of patients who underwent BCS with AAF had unacceptable cosmetic results. In particular, the percentage of unacceptable cases increased when AAF was used in patients with medium-sized breasts. In addition, unacceptable outcomes were observed more frequently in older patients.

To address these limitations, we introduced a modified AAF technique that optionally incorporates the Ochikomanai method, specifically designed for breast cancer cases in the lower region. As shown in Table 3, the modified AAF demonstrated significantly better cosmetic outcomes than the standard AAF, particularly in older patients and those with larger breasts. Our previous study indicated that standard AAF resulted in a high percentage of unacceptable outcomes (26.8%), particularly in older patients [9]. In contrast, the present study found that the modified AAF reduced the unacceptable rate to 5%, suggesting that this technique is particularly beneficial for this patient population. One possible reason for this is that modified AAF requires less extensive subcutaneous dissection than standard AAF, preserves natural breast tension, and reduces the risk of skin depression. In addition, the Ochikomanai method further contributes to improved contours by preventing excess skin from sinking into the resection site. These findings indicate that modified AAF is a valuable alternative to standard AAF, particularly in populations at an increased risk of poor aesthetic outcomes.

The modified AAF technique is particularly suitable for tumors located in the lateral and caudal regions, with an estimated excised volume of approximately 25% of the total breast volume. Unlike conventional AAF, this technique showed good outcomes, even in medium- and large-breasted patients. There were no strict exclusion criteria based on comorbidities, such as diabetes or advanced age, although the procedure was rarely applied to tumors in the central or upper inner regions. In particular, among patients with medium-sized or larger breasts, 57% who underwent BCS with AAF had unacceptable outcomes compared to only 5% of those who underwent BCS with modified AAF. Among patients who underwent BCS with AAF, the average age of those considered to have unacceptable outcomes was higher than that of those with acceptable outcomes, although there were no significant differences. In contrast, among the patients who underwent BCS with modified AAF, there were no marked age differences between those with acceptable and unacceptable outcomes. The Ochikomanai method was applied in 36% of the cases, primarily in patients with excess skin remaining after tumor resection. Patients with a larger breast volume or thin skin were more likely to develop postoperative contour depression those with smaller breasts or thicker skin, which was effectively prevented using this technique. This method allows for a more natural transition between the neo-IMF and the surrounding tissue, contributing to better aesthetic outcomes.

We identified five key factors that likely contributed to the favorable outcomes observed with BCS using the modified AAF, both with and without the Ochikomanai method, particularly in patients with medium to large breasts and older age. First, the reduced subcutaneous dissection area helps preserve the natural tension in the lower breast, as no additional subcutaneous reshaping is necessary. The second is suture fixation of the neo-IMF and upper mammary gland. This suture fixation allows simultaneous elevation of the neo-IMF and descent of the cranial mammary gland, including the nipple-areolar complex. We believe that when the NAC descends with cranial breast tissue, the risk of NAC cranial deviation is reduced. Third, the Ochikomanai method addresses the issue of excess skin depression, which is more prevalent in older patients with thin skin and adipose-rich breast tissues. In such cases, the method effectively prevents the depression of excess skin in the resection defect, thus enhancing cosmetic outcomes. The fourth factor is the excision volume. When BCS was performed with the modified AAF, the average percentage of excision volume compared with the total breast volume was lower than that when BCS was performed with the AAF.

We consider the reason for the reduced resection volume to be the expanded indications for BCS with a modified AAF. Although not in comparison to AAF, modified AAF is a simpler procedure than AAF, requiring less time for surgery, with only minor complications that can be improved with conservative treatment. The modified AAF is less stressful for both patients and surgeons because the procedure is simpler. Therefore, the indications for BCS with modified AAF were expanded to include not only cases with large resection volumes that are prone to deformity but also those with small resection volumes. The final potential reason is the improvement in the surgeons’ skills. We compared the results of BCS with those of AAF and modified AAF performed at the same hospital. Both the AAF and modified AAF techniques include IMF reshaping by inserting absorbable sutures into the neo-IMF and elevating it. Surgeons may become more accustomed to the technique and improve their skills, which may have improved the cosmetic results of BCS using the modified AAF.

Patient feedback highlighted high satisfaction across most of the evaluated categories, with the exception of early postoperative physical limitations. Approximately one-third of the patients reported experiencing significant physical limitations shortly after surgery, which typically resolved within six months. Immediately after surgery, absorbable sutures can entangle the IMF, and patients often complain of skin tightness. We believe that it is important to explain that skin tightness may be felt immediately after surgery and then loosened as the absorbable sutures are absorbed.

Several limitations associated with the present study warrant mention. This single-center study in Japan may have limited generalizability. There was a selection bias because fewer patients with modified AAF had tumors in the inner portion of the breast. The modified AAF technique uses the skin and subcutaneous tissue of the upper abdomen and lateral chest as flaps, and advancing the lateral thoracic tissue to the outer portion is easy. Therefore, we selected patients with good indications for tumors in the outer portion. Whether or not additional patients with tumors in the inner portion of the lesions would necessarily have the same results as those in this study is unclear. As this was a retrospective study, the criteria for selecting cases of BCS with modified AAF were not clearly defined, which may have caused selection bias. The use of the BREAST-Q to conduct the patient evaluations has become increasingly common. However, because the BREAST-Q has a large number of questions, it can reduce the response rate of the patients. Therefore, we developed our original questionnaire using simple questions to increase patient response rates. All patients who underwent BCS with the modified AAF were evaluated using our original questionnaire, which may make it difficult to generalize the assessment.

## Conclusions

In this study, BCS using the modified AAF with or without the Ochikomanai method was simpler and, therefore, less stressful than the AAF for both patients and surgeons in terms of operating time, blood loss, and complications. Both the cosmetic results and patient satisfaction were satisfactory in our study. We believe that BCS using a modified AAF, with or without the Ochikomanai method, is a useful technique.
